# Fulminant Transfusion-Associated Hepatitis E Virus Infection Despite Screening, England, 2016–2020

**DOI:** 10.3201/eid2809.220487

**Published:** 2022-09

**Authors:** Heli Harvala, Claire Reynolds, Su Brailsford, Katy Davison

**Affiliations:** University College of London, London, UK (H. Harvala);; NHS Blood and Transplant, London (H. Harvala, C. Reynolds, S. Brailsford, K. Davison);; UK Health Security Agency, London (K. Davison)

**Keywords:** hepatitis E virus, blood donor, platelets, hepatitis, transmission, viruses, England, HEV, enteric infections

## Abstract

In England, all blood donations are screened in pools of 24 by nucleic acid test (NAT) for hepatitis E virus (HEV) RNA. During 2016–2020, this screening successfully identified and intercepted 1,727 RNA-positive donations. However, review of previous donations from infected platelet donors identified 9 donations in which HEV RNA detection was missed, of which 2 resulted in confirmed transmission: 1 infection resolved with ribavirin treatment, and 1 proceeded to fatal multiorgan failure within a month from infection. Residual risk calculations predict that over the 5-year study period, HEV RNA detection was missed by minipool NAT in 12–23 platelet and 177–354 whole-blood donations, but transmission risk remains undetermined. Although screening has been able to largely eliminate infectious HEV from the blood supply in England, missed detection of low levels of HEV RNA in donated blood can lead to a severe, even fulminant, infection in recipients and could be prevented by more sensitive screening.

Hepatitis E virus (HEV) is a nonenveloped enteric virus, classified in the genus *Orthohepevirus*, family *Hepeviridae* (*1*). HEV is divided into 8 genotypes that differ substantially in their host ranges and zoonotic capacities. Genotypes 1 and 2 typically infect humans and have been associated with large waterborne outbreaks of infections in developing countries. By contrast, genotypes 3 and 4 are endemic among domestic pigs, wild boar, and rabbits; most pigs reared for pork production in Europe have been infected with genotype 3 (*2*), and HEV RNA is detected in 40% of farmed pig fecal samples (*3*). Humans are increasingly exposed to HEV in developed countries through consumption of uncooked processed or cured pork products, typically with genotype 3 in Europe, North America, and Japan and with genotype 4 in China and other parts of Southeast Asia (*2*,*4*–*7*). Seroprevalence of HEV antibodies, indicative of past exposure and infection, are in the range of 5%–13% in the United Kingdom (*8*–*10*) and in the low-to-middle range of incidence estimated for other countries in Europe (*11*–*13*). Recent increases in human infections (*14*) are undoubtedly the direct consequence of increasing circulation of HEV in farmed pigs; 20% of pigs have active infection at time of slaughter, posing universal human exposure risk at time of slaughter (*15*).

Ongoing and increasing HEV endemicity among farmed pigs creates downstream effects for human blood transfusion from infected blood donors. HEV infections are associated with variable periods (3–5 weeks) of often intense viremia (*16*) during acute infections that may transmit infections to blood recipients (*17*,*18*). Measured frequencies of RNA as an indicator of viremia in blood donors are remarkably high, ≈1 detection/2,000 donations tested to 1 detection/3,000 donations according to several studies of blood donors in different countries in western Europe (*17*,*19*,*20*) and as high as 1 detection/762 donations in the Netherlands (*21*) and 1 detection/157 donations in Italy (*13*). Several studies have documented transmission of HEV infections to blood recipients (*16*,*17*,*20*,*22*), typically from donations that have higher viral loads and are seronegative for HEV antibodies and from blood components with higher residual plasma volumes, such as platelets (*2*,*17*). A proposed threshold by which infectious and noninfectious donations could be differentiated was 2 × 10^4^ IU of HEV RNA (*23*).

Although HEV infections are typically mild and self-resolving, those in immunocompromised blood and platelet recipients may persist and induce rapidly progressive liver disease and frequently cause death (*24*–*27*). The potential for severe disease outcomes in recipients of blood components has led to detailed modeling-based investigations of HEV RNA screening for blood and platelet donors (*28*). Only the United Kingdom, Ireland, and the Netherlands now perform universal donor screening; for immunocompromised patients, many blood services in Germany, France, and Switzerland also perform selective screening for components. Many countries, notably the United States, have elected not to screen on the basis of low risk and cost-effectiveness (*29*). Among blood services that do screen for HEV RNA, the lack of international consensus for screening requirements has introduced further variability in the pool sizes used for screening; larger pools reduce costs but also sensitivity. A number of modelling studies have attempted to estimate residual infectivity risk by using different pool sizes and assay formats for HEV RNA testing (*30*–*33*), albeit with conclusions tempered by ongoing uncertainty over the upper limit of viral loads that are noninfectious.

In the United Kingdom, transfusion complications are monitored by the Serious Hazards of Transfusion (SHOT) hemovigilance group (https://www.shotuk.org/shot-reports). However, reported cases of HEV transmission may underestimate their true extent because infections may be unsuspected and underdiagnosed or not linked to blood component transfusion in patients in whom complications may develop.

With this study, we aimed to describe the burden of HEV among blood donors, determine the risk for nondetection of HEV, and reassess the extent and effect of transfusion-transmitted infections in the presence of a national screening programme. Signed consent was obtained from each donor before blood collection, including consent for NHS Blood and Transfusion (NHSBT) to use their data for clinical audit to assess and improve our services, as well as to increase our knowledge of the donor population.

## Methods

### Study Population

We used surveillance data collected by the joint NHSBT/UK Health Security Agency (UKHSA) Epidemiology Unit from all HEV RNA–positive apheresis and whole-blood donors identified in England from March 1, 2016, through December 31, 2020. We also used the total number of donations tested in both categories within that period.

### HEV Testing of Donated Blood

We tested all apheresis donations collected in England during the study period for HEV RNA. On April 10, 2017, testing of whole-blood donations changed from selective to universal, for reasons of operational simplicity and cost-effectiveness. We performed HEV RNA testing in pools of 24 donations (cobas MPX for use on the 6800/8800 systems; Roche Diagnostics, https://www.roche.com). A reported 95% limit of detection was 18.6 IU/mL, translating to 446 IU/mL at the individual donation level when tested in pools of 24. We resolved reactive pools to individual HEV RNA–containing donations by using the same assay and subjected individual samples to confirmatory HEV testing, including serology and molecular investigations at the Microbiology Services Laboratory (NHSBT, Colindale, UK) as described previously (*34*). Donations in which HEV RNA was detected at screening were removed from the clinical supply, and the donors were followed up clinically (informed and advised to contact their general practitioner if unwell) (*34*).

### Archived Samples and Testing of Transfusion Recipient when Indicated

NHSBT archived 900 μL plasma from each blood donation for a minimum of 3 years. We requested the archived plasma sample from the most recent HEV RNA–negative donation for individual HEV RNA and antibody testing for all apheresis donors whose subsequent donation was HEV RNA positive. If HEV RNA was detected, we also retrieved the next most recent archive sample for testing. During March 2016–June 2018, hospitals that received the HEV RNA–positive blood components were informed of potential risks and advised to consider appropriate actions. Since July 2018, clinical teams have followed up with transfusion recipients, and testing for HEV RNA and IgG for up to 6 months after transfusion has been recommended.

### Transfusion-Transmitted Infections

The NHSBT/UKHSA Epidemiology Unit collated data on all suspected transfusion-transmitted HEV infections reported to, and investigated by, NHSBT during the 5-year study period and reported them to SHOT. The outcomes of the investigations were determined to be confirmed, probable, or not transfusion-transmitted infection. We did not consider transmissions that had occurred before the introduction of HEV screening.

### Residual Risk Calculations

To estimate residual risk of not detecting HEV RNA in apheresis and whole-blood donations, we applied the traditional window period method to HEV testing data. We assume that nondetection resulted from proximity to initial infection in a window period of HEV NAT screening. The number of apheresis and whole-blood donations tested and found positive for HEV RNA were available from NHSBT/UKHSA epidemiology surveillance for March 2016 through December 2020.

We assumed incidence to be equal to the rate of HEV RNA positivity among donors. To estimate risk, we multiplied incidence by the duration of the window period in years for HEV NAT by using 0.019 years (corresponding to 7 days) and 0.038 years (corresponding to 14 days). These parameters were based on expert opinion in the absence of any published values. We calculated risk for apheresis and whole-blood donations by year per million donations tested. To derive an approximate number of undetected HEV RNA-positive donations, we extrapolated risks to the estimated number of apheresis and whole-blood donations each year.

## Results

### Detection of HEV RNA in Donated Blood

From March 1, 2016, through December 31, 2020, a total of 6,297,904 blood donations, including 350,323 apheresis and 5,947,581 whole-blood donations, were screened for HEV RNA in England. HEV RNA was detected and infection confirmed in all 1,727 initially RNA-reactive blood donations collected from 1,727 blood donors, of which 107 were apheresis and 1,620 whole-blood donors (Table 1). Most blood donors (1,559,517) were screened in 2018, and most HEV RNA-positive donations (470) were identified in 2019. Overall, HEV RNA was detected in 1 of every 2,000 donors (varying from 1/1,258 donors in 2016 to 1/2,591 donors in 2017) and in 1 of every 3,647 donations (varying from 1/1,258 donations in 2016 to 1/2,591 donations in 2017).

**Table 1:** Number of donations and donors tested for hepatitis E virus RNA, number of positive results, detection rate, and incidence rate for apheresis and whole-blood donors, England, March 2016–December 2020

Donation type, year	Donations	Donors	Positive	Detection rate, no. HEV RNA detections/1 million donations	Incidence rate, HEV RNA–positive samples/1 million donors
Apheresis					
2016	69,605	14,952	24	344.8	1,966.5
2017	74,422	15,987	20	268.7	1,532.6
2018	70,709	15,189	24	339.4	1,935.8
2019	68,907	15,450	15	217.7	1,241.5
2020	66,680	13,593	24	359.9	2,052.7
Total	350,323	75,170	107	305.4	1,741.9
Whole blood					
2016	413,153	234,141	174	421.2	2,401.9
2017	1,256,503	712,083	261	207.7	1,184.6
2018	1,488,808	843,734	351	235.8	1,344.6
2019	1,450,628	825,363	455	313.7	1,788.8
2020	1,338,489	769,420	379	283.2	1,614.9
Total	5,947,581	3,384,741	1,620	272.4	1,553.4

Viral loads were available for 1,200 HEV RNA–positive donations. The geometric mean viral load was 998 IU/mL (range 1–8.7 × 10^6^ IU/mL); viral load was below the theoretical 95% detection limit of screening assay used in 35.8% of donations. Of note, <100 IU/mL of HEV RNA was detected in 17% of donations (Figure). All donations that were retrospectively shown to contain HEV RNA had a low viral load, below the level of reliable quantification (37 IU/mL).

**Figure:**
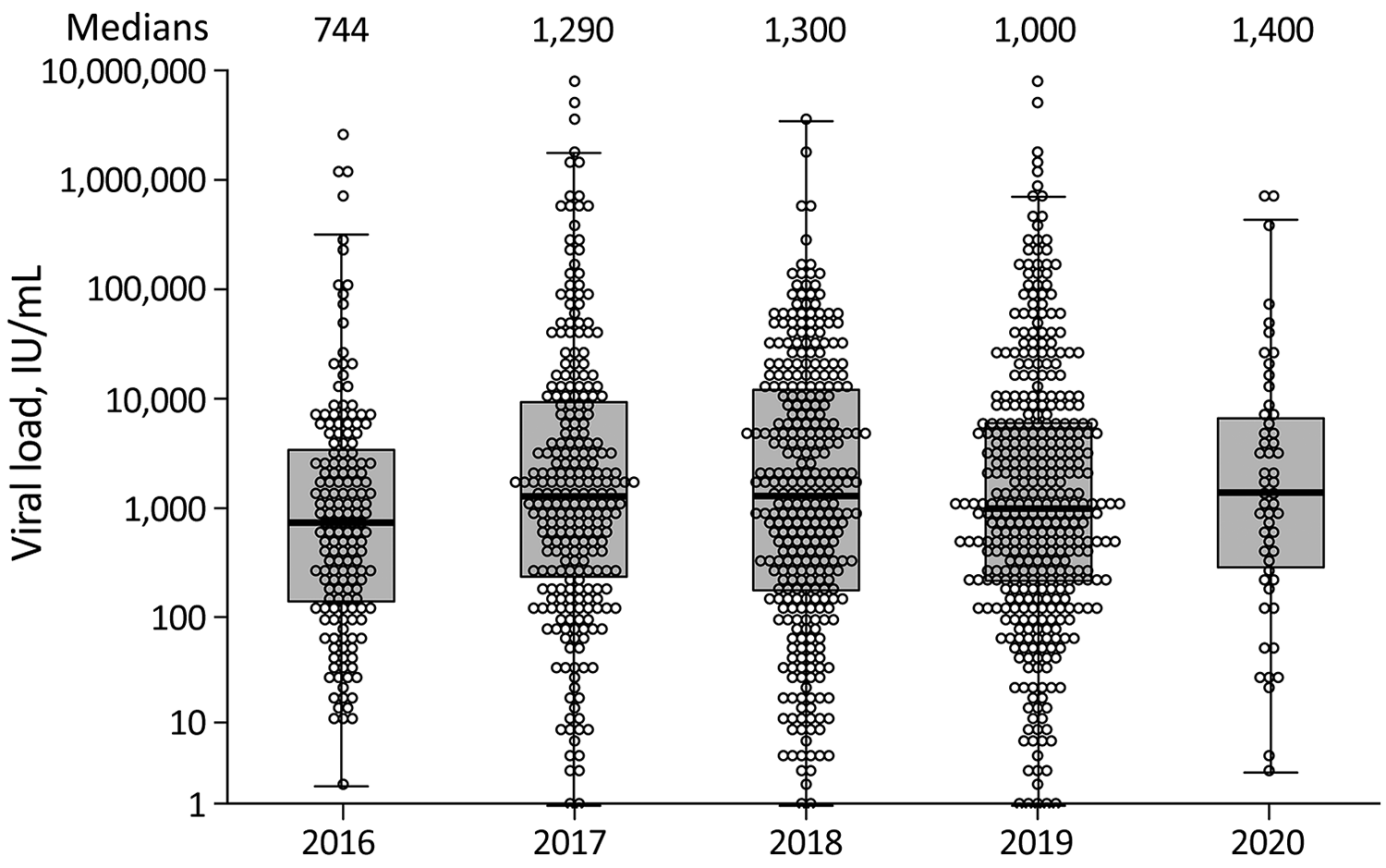
Hepatitis E virus viral loads in 1,200 individual blood donors in England, 2016–2020. Median viral loads were comparable over the study period. Circles indicate individual donors; horizontal lines within boxes and numbers above plots indicate medians; boxes indicate first and third quartile values; whiskers indicate lowest and highest data points.

### Residual Risk for HEV Nondetection

The annual HEV incidence and estimated risk for nondetection fluctuated each year for apheresis and whole-blood donors (Table 2). For apheresis donors, estimates were highest in 2020 (incidence 2,052.7 and risk for nondetection 39.34 per 1 million donations tested), whereas estimates for whole-blood donors were highest when screening was first introduced in 2016 (incidence 2,401.9 and risk for nondetection 46.03 per 1 million donations tested). Risk for both groups of donors increased 2-fold if a window period of 14 days instead of 7 days was used. During the study period, the estimated risk, with a 7-day window period, predicted that 12 apheresis and up to 177 whole-blood donations positive for HEV RNA were not detected (Table 3); that prediction increased to 24 apheresis and 354 whole-blood donations positive when a 14-day window period was assumed.

**Table 2:** Incidence of hepatitis E virus RNA positive samples and estimated risk for nondetection, with 7-day window period risk for apheresis and whole blood donors. England, March 2016–December 2020

Donation type, year	Incidence, HEV RNA–positive samples/1 million donors	Risk per 1 million if window period 7 days (95% CI)
Apheresis		
2016	1,966.5	37.69 (24.15–56.07)
2017	1,532.6	29.37 (17.94–45.36)
2018	1,935.8	37.10 (23.77–55.20)
2019	1,241.5	23.79 (13.32–39.24)
2020	2,052.7	39.34 (25.21–58.53)
Whole blood		
2016	2,401.9	46.03 (39.45–53.40)
2017	1,184.6	22.70 (20.03–25.63)
2018	1,344.6	25.77 (23.14–28.61)
2019	1,788.8	34.28 (31.21–37.58)
2020	1,614.9	30.95 (27.91–34.23)

**Table 3:** Estimated number of hepatitis E virus RNA–positive donors not detected, according to 7-day window period risk for apheresis and whole blood donors, England, March 2016–December 2020

Donation type, year	Donations tested	No. not detected if window period 7 days (95% CI)
Apheresis		
2016	69,605	2.62 (1.68–3.90)
2017	74,422	2.19 (1.34–3.38)
2018	70,709	2.62 (1.68–3.90)
2019	68,907	1.64 (0.92–2.70)
2020	66,680	2.62 (1.68–3.90)
Total	350,323	11.70 (7.30–17.79)
Whole blood		
2016	413,153	19.02 (16.30–22.06)
2017	1,256,503	28.53 (25.17–32.21)
2018	1,488,808	38.36 (34.46–42.59)
2019	1,450,628	49.73 (45.27–54.52)
2020	1,338,489	41.42 (37.36–45.81)
Total	5,947,581	177.07 (158.55–197.19)

### HEV Lookback

During the 5-year study period, a total of 107 HEV RNA–positive apheresis platelet donations were identified (Table 4). Retrospective individual HEV RNA testing of donors’ 98 previous donations identified 9 HEV RNA–positive donations that were undetected by screening because of low viral loads (<37 IU/mL) and supplied for clinical use. Retrospective investigations included identification and follow-up of the 18 recipients of the platelet components produced from the indicated donations (Table 5). All components were traced; no data were received back from 8 recipients, 3 had subsequently died, and sufficient follow-up data were received for the remaining 7. Follow-up testing excluded HEV infection in 5 recipients (all were HEV IgG and RNA negative 6 months after transfusion) and confirmed HEV infection in 2 recipients (1 in 2018 and 1 in 2019; both were HEV RNA positive). Further investigations, including sequence analysis, confirmed that both recipients had acquired their infection via transfusion.

**Table 4:** Nondetection of HEV RNA based on retrospective investigations for apheresis donors, in which indicated donations are individually retested for HEV RNA, England, March 2016 to–December 2020

Year(s)	Screening			Previous donations		
	No. apheresis donations screened	No. HEV RNA–positive donations		No. retrospectively tested	No. HEV RNA–positive donations identified by retesting	No. components produced and supplied for clinical use
2016–2017	144,027	44		35	2	4
2018	70,709	24		24	3	6
2019	68,907	15		14	2	4
2020	66,680	24		*25*	2	4
Total	350,323	107		98	9	18

**Table 5:** Details of lookback investigations into 9 HEV RNA positive platelet donations supplied for clinical use in England, March 2016 to December 2020*

Donation no.	HEV viral load, IU/mL†	Components	Recipient details	Recipient follow-up testing results
Donation 1	4	Platelet 1	Hospitals informed, no data back	NA
		Platelet 2		
Donation 2	8	Platelet 1	Hospitals informed, no data back	NA
		Platelet 2		
Donation 3	<37	Platelet 1	Hospitals informed, no data back	NA
		Platelet 2		
Donation 4	<37	Platelet 1	Hospitals responded, no evidence of transmission	NA
		Platelet 2		
Donation 5	37	Platelet 1	M, 50–60 years, B-cell lymphoma	Confirmed transmission
		Platelet 2	F, 20-30 y, sepsis	Deceased
Donation 6	37	Platelet 1	F, 60–70 y, aplastic anemia	Tested negative
		Platelet 2	M, 50–60 y, lymphoma	Tested negative
Donation 7	4.5	Platelet 1	M, 40–50 y, aplastic anemia	Confirmed transmission
		Platelet 2	F, 50–60 y, vascular syndrome	Tested negative
Donation 8	10	Platelet 1	M, 60–70 y, aplastic anemia	Tested negative
		Platelet 2	M, <10 y, cancer	Deceased
Donation 9	3	Platelet 1	F, 40–50 y, myelodysplastic syndrome	Tested negative
		Platelet 2	M, 40–50 y, acute myeloid leukemia	Deceased

*Each donation was tested for HEV RNA and divided into 2 platelet packs. †Estimated viral loads given where available. HEV, hepatitis E virus; NA, not applicable.

### Confirmed HEV Transmission in 2018

Retrospective individual PCR testing detected a low level (37 IU/mL) of HEV RNA in a platelet donation. One recipient was a young patient with sepsis, who died of an underlying condition 2 days after transfusion. The other was a hematology patient who had completed treatment for B-cell lymphoma. He became viremic 8 weeks after transfusion and was immediately placed under the care of the hepatology team. He received ribavirin, and ≈6 months later, the HEV infection cleared. Identical HEV sequences obtained from donor and recipient samples confirmed the blood transfusion as a source of this HEV infection.

### Confirmed HEV Transmission in 2019

The index apheresis platelet donation contained low levels of HEV RNA (<37 IU/mL). A male patient, 40–50 years of age, with aplastic anemia and portal hypertension received the first unit of apheresis platelets in September 2019. At the time of HEV diagnosis in early November 2019 (sample taken at our advice), he was clinically well. He was monitored carefully and remained stable with unchanged liver function test results until mid-November, when his viral load peaked at 29,200,000 IU/mL and a strong antibody response against HEV developed, coinciding with a sudden increase in bilirubin and aspartate aminotransferase levels. Treatment with ribavirin was initiated, but his liver and renal function continued to deteriorate, leading to acute hepatitis with kidney failure, and he died at the end of November. Transmission was confirmed by sequence identity of the infecting HEV strains and of the subsequent apheresis donation (viral load of 4,900 IU/mL), collected 3 weeks after the index donation.

The other recipient of platelets from this donation had vascular type Ehlers-Danlos syndrome and was followed up for 6 months. HEV infection was excluded by both serology and molecular testing.

### Transfusion-Transmitted Infections

In England, all suspected transfusion-transmitted infections investigated by NHSBT are reported to the NHSBT/UKHSA Epidemiology Unit and to SHOT. During 2016–2020, NHSBT investigated and reported 6 cases of possible transfusion-transmitted HEV. Transfusion-transmitted infection was not considered for 3 cases, was confirmed for 2 cases, and was probable for the remaining case, for which a red blood cell transfusion was shown to be the probable source of the recipient’s HEV infection. The recipient was a multitransfused young adult with aplastic anemia and Turner syndrome; infection cleared after ribavirin treatment, but the virus in the donor could not be typed because of low levels of viremia.

## Discussion

To mitigate the risk for transfusion-transmitted chronic HEV infection in immunocompromised patients in the United Kingdom, blood donation screening for HEV RNA was introduced in March 2016. Since then, through screening of >6.2 million blood donations for HEV RNA in England, 1,727 HEV RNA–positive donations have been removed from the blood supply and only 2 cases of transfusion-transmitted HEV infection have been identified. Although these findings suggest that the pooled screening strategy has been able to largely eliminate the infectious HEV from the blood supply in England, our residual risk calculations argue against that conclusion.

Although individual NAT represents the most effective screening method for identifying HEV RNA in donations, including those with the lowest level of HEV RNA, our data suggest that pooled NAT performs better than the theoretically calculated sensitivity would indicate. Almost one third of identified HEV RNA–positive donations had a viral load below the theoretical assay sensitivity of 446 IU/mL calculated for individual donors (430/1,200, 36%), and approximately half of those had a viral load of <100 IU/mL (206/1,200, 17%). These data are comparable to those obtained by individual NAT screening with an increased sensitivity of 7.89 IU/mL (Grifols, https://www.grifols.com) in Ireland, where 37.5% of HEV RNA–containing blood donations had a viral load of <100 IU/mL (*18*).

Consistent with those data, during our 5-year period of pooled screening, missed HEV RNA detection in donations retrospectively identified low levels of HEV RNA (<37 IU/mL). Although pooled HEV screening is generally effective for identifying donations with low-level viremia, the process repeatedly missed donations with HEV RNA <37 IU/mL. Similar findings were obtained in Germany, where all HEV RNA–positive donations exclusively identified by individual NAT screening had viral loads of <25 IU/mL (*32*,*33*).

When we further calculated the residual risk for HEV RNA nondetection on the basis of 7-day and 14-day window periods, the estimated risk predicted that 12–24 apheresis platelet donations and 177–354 whole-blood donations positive for HEV RNA would not have been detected over the 5-year study period. Despite uncertainty in the parameters, this predicted risk is similar to the true risk demonstrated so far for apheresis donations: during 2016–2020, initial pooled screening did not detect HEV RNA positivity in 9 apheresis donations.

Although the model predicted that the screening is missing HEV RNA in a much larger number of whole-blood donations, these missed detections do not directly equate to transmission risk. Infectious HEV is considered to partition into the plasma component of a donation; the residual plasma volume is substantially higher in platelet (180 mL) than in red blood cell (5 mL) donations. The 7-fold lower residual plasma volume in red blood cell donations may therefore reduce transmission risk from this component. Indeed, the estimated maximum dose of HEV RNA in red blood cell donations containing low levels of HEV RNA would probably remain at <1,000 IU of HEV RNA (37 IU/mL × 25 mL = 925 IU), in contrast to the level predicted for platelet donations (37 IU/mL × 180 mL = 6,660 IU).

The minimum infectious dose of HEV RNA leading to an infection in the transfusion recipient remains unknown. Earlier studies suggested that ≈20,000 IU of HEV RNA would be required for efficient transmission (*23*,*35*). Since then, lower amounts of HEV RNA have been occasionally associated with virus transmission (*36*). So far, the lowest amount of HEV RNA leading to transmission is 7,056 IU; transmission occurred via apheresis platelet transfusion and resulted in chronic HEV infection in an immunosuppressed recipient (*20*). Although we could not determine the exact infectious doses linked to the 2 cases of HEV transmission, we can estimate that doses leading to an infection were <6,660 IU of HEV RNA. Both cases were identified in our study via the active lookback investigation in which doctors caring for these recipients were contacted and the importance of HEV RNA testing even without the symptoms was explained. Both recipients were tested 8 weeks after transfusion and were completely asymptomatic at the time of testing. Even if HEV had been considered as a diagnosis when additional symptoms developed, it is possible that HEV infection would not have been linked to the previous transfusions. Our findings indicate the value of active lookback investigations in cases for which infections may easily be missed or unsuspected clinically. This approach estimates the true minimum infectious dose of HEV RNA required for transmission via blood transfusion.

HEV RNA has traditionally been considered to be largely present in plasma; hence, plasma volume determines the risk for transmission. However, the potential sequestration of HEV virions by platelets should also be considered as contributors to HEV transmission risk from this blood component. Although the main function of platelets is to maintain vascular integrity, they also play roles in viral immune responses. For example, platelets express many receptors, including integrins, that mediate virus attachment and can hence bind to free virus and present it to neutrophils (*37*). Integrins also play a key role for cellular attachment and entry of HEV (*38*). Given the association of HEV transmission with platelet transfusions, whether platelets do indeed bind HEV and contribute to transmission risk independently from HEV circulating in plasma is an additional parameter. Although this platelet-bound virus is probably controlled within an immunocompetent donor host, it could lead to a more severe infection in recipients with suboptimal platelet and neutrophil reservoirs. This consequence could explain the severe outcome in aplastic anemia patients in whom the bone marrow fails to produce enough blood cells because the normal hematopoietic stem cells are replaced by abnormal cells.

HEV genotype 3 can cause a chronic infection and occasionally lead to cirrhosis or even liver failure in immunosuppressed patients and those with underlying chronic liver disease (*24*–*27*). In our study, chronic HEV infection developed in 1 patient with B-cell lymphoma and the HEV infection was successfully treated with ribavirin. However, acute hepatitis with kidney failure developed in the other patient, who had aplastic anemia and died within a month of acquiring the HEV infection via blood transfusion. This outcome was unexpected because aplastic anemia has not previously been considered to be a particular risk factor for HEV infection. Although it might have been a coincidence that the third probable HEV transmission event investigated in England in 2019 was also associated with a recipient with aplastic anemia, those results prompted us to review fulminant cases of transfusion-transmitted HEV infection in patients with aplastic anemia reported in the literature. Of note, 2 patients with aplastic anemia acquired HEV infection via blood transfusion and the cases were reported; both patients died, 1 with sepsis and 1 with cardiac issues (*17*). These data led to the possibility that patients with aplastic anemia might specifically be at higher risk for transfusion-transmitted HEV infection, underlying the value of active surveillance looking for unusual manifestations in blood recipients beyond the typical hepatitis.

In conclusion, the pooled screening strategy has been able to largely eliminate infectious HEV from the blood supply in England because only 2 confirmed transfusion-transmitted HEV infections have been reported since the screening began in 2017. However, 2 cases are probably an underestimate because the analysis of HEV transmissions was based on a small number of donations in which HEV RNA was known to have been missed by pooled screening and even fewer recipients who were available for active follow-up. Further residual risk calculations predict that very low levels of HEV RNA were missed in up to 24 apheresis and 354 whole-blood donations during the study period. This information is especially useful now because our results show that even these low levels of HEV RNA can lead to a severe, even fulminant, infection in the recipient.
